# Temporo-spatial cell-cycle kinetics in HeLa cells irradiated by Ir-192 high dose-rate remote afterloading system (HDR-RALS)

**DOI:** 10.1186/s13014-016-0669-8

**Published:** 2016-07-29

**Authors:** Taito Asahina, Atsushi Kaida, Tatsuaki Goto, Ryo-Ichi Yoshimura, Keisuke Sasai, Masahiko Miura

**Affiliations:** 1 Department of Radiation Oncology, Juntendo University, 3-1-3 Hongo, Bunkyo-ku, Tokyo, 113-8431 Japan; 2 Department of Oral Radiation Oncology, Department of Oral Health Science, Graduate School of Medical and Dental Sciences, Tokyo Medical and Dental University, 1-5-45 Yushima, Bunkyo-ku, Tokyo, 113-8549 Japan; 3 Department of Radiation Therapeutics and Oncology, Division of Maxillofacial and Neck Reconstruction, Graduate School of Medical and Dental Sciences, Tokyo Medical and Dental University, 1-5-45 Yushima, Bunkyo-ku, Tokyo, 113-8549 Japan

**Keywords:** Cervical cancer, Intracavitary irradiation, Fucci, G2 arrest, Ir-192, HDR-RALS

## Abstract

**Background:**

Intracavitary irradiation plays a pivotal role in definitive radiotherapy for cervical cancer, and the Ir-192 high dose-rate remote afterloading system (HDR-RALS) is often used for this purpose. Under this condition, tumor tissues receive remarkably different absorption doses, with a steep gradient, depending on distance from the radiation source. To obtain temporo-spatial information regarding cell-cycle kinetics in cervical cancer following irradiation by Ir-192 HDR-RALS, we examined HeLa cells expressing the fluorescence ubiquitination-based cell cycle indicator (Fucci), which allowed us to visualize cell-cycle progression.

**Methods:**

HeLa-Fucci cells, which emit red and green fluorescence in G1 and S/G2/M phases, respectively, were grown on 35-mm dishes and irradiated by Ir-192 HDR-RALS under normoxic and hypoxic conditions. A 6 French (Fr) catheter was used as an applicator. A radiation dose of 6 Gy was prescribed at hypothetical treatment point A, located 20 mm from the radiation source. Changes in Fucci fluorescence after irradiation were visualized for cells from 5 to 20 mm from the Ir-192 source. Several indices, including first green phase duration after irradiation (FGPD), were measured by analysis of time-lapse images.

**Results:**

Cells located 5 to 20 mm from the Ir-192 source became green, reflecting arrest in G2, in a similar manner up to 12 h after irradiation; at more distant positions, however, cells were gradually released from the G2 arrest and became red. This could be explained by the observation that the FGPD was longer for cells closer to the radiation source. Detailed observation revealed that FGPD was significantly longer in cells irradiated in the green phase than in the red phase at positions closer to the Ir-192 source. Unexpectedly, the FGPD was significantly longer after irradiation under hypoxia than normoxia, due in large part to the elongation of FGPD in cells irradiated in the red phase.

**Conclusion:**

Using HeLa-Fucci cells, we obtained the first temporo-spatial information about cell-cycle kinetics following irradiation by Ir-192 HDR-RALS. Our findings suggest that the potentially surviving hypoxic cells, especially those arising from positions around point A, exhibit different cell-cycle kinetics from normoxic cells destined to be eradicated.

**Electronic supplementary material:**

The online version of this article (doi:10.1186/s13014-016-0669-8) contains supplementary material, which is available to authorized users.

## Background

A combination of external and intracavitary irradiation is used as a definitive radiotherapy for treatment of cervical cancer [1–3]. Intracavitary irradiation using a high dose-rate radiation source, such as Ir-192, has been applied in the form of a remote afterloading system (RALS) [2, 3]. The prescribed dose is usually ~6 Gy per fraction at point A, 2 cm from the center axis of the uterus [4]. Although the dose at this point is considered to be an index of tumor dose, it is widely recognized that tumor tissues receive remarkably different doses, with a steep gradient, depending on distance from the radiation source. We reasoned that radioresponses of tumor cells, including cell-cycle kinetics, following such an intracavitary irradiation should differ markedly depending on distance from the radiation source.

Following DNA damage, cell-cycle progression stops at the G1/S and G2/M checkpoints [5]. The former checkpoint is dependent on p53 function, whereas the latter is not [6, 7]. Therefore, tumor cells with p53 gene mutations or human papilloma virus (HPV) infection only activate the G2/M checkpoint following irradiation, resulting in G2 arrest. Flow-cytometric analysis using DNA content as a marker has been used to detect radiation-induced G2 arrest [8]; however, because this method requires preparation of single cells and fixation, spatial information is lost, and temporal information must be obtained from different cell populations.

To address these issues, we used the fluorescent ubiquitination-based cell cycle indicator (Fucci) system, in which cells emit red and green fluorescence in G1 and S/G2/M phases, respectively. The Fucci system allowed us to visualize cell-cycle progression in HeLa cells, which lack p53 function due to HPV infection [9]. In a previous study using time-lapse imaging, we showed that the fluorescence kinetics in HeLa-Fucci cells perfectly reflect radiation-induced G2 arrest kinetics [10, 11]. In this study, taking further advantage of this system, we attempted to visualize cell-cycle kinetics in vitro, as a function of the distance from the radiation source, after irradiation with an Ir-192 HDR-RALS under normoxic and hypoxic conditions. Furthermore, we determined the effect of cell-cycle phase at irradiation under both conditions.

## Methods

### Cell line and culture conditions

HeLa cells expressing the Fucci system (HeLa-Fucci) were provided by the RIKEN BRC through the National Bio-Resource Project of MEXT, Japan. Cells were maintained in DMEM (Sigma-Aldrich, St. Louis, MO, USA) supplemented with 100 units/ml penicillin, 100 μg/ml streptomycin, and 10 % fetal bovine serum at 37 °C in a 5 % CO_2_ humidified atmosphere.

### Irradiation by Ir-192 HDR-RALS

**Fig. 1:**
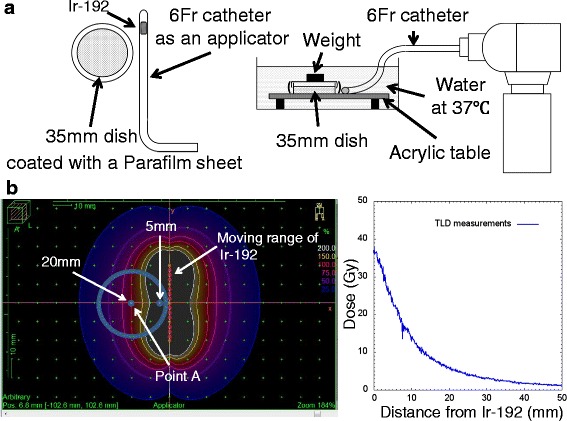
Experimental conditions for irradiation by Ir-192 HDR-RALS. **a** Schematic diagram of the experimental conditions. A 35-mm glass-bottom dish containing cultured HeLa-Fucci cells was coated with a Parafilm sheet. The dish and catheter were fixed on the table in the same plane using adhesive tape. Irradiation was performed in a water bath. **b** Dose distribution under these experimental conditions. Left panel: Isodose curves. Radiation dose of 6 Gy was prescribed at hypothetical treatment point A, 20 mm away from the Ir-192 source (*dotted line*), according to a simulation by the treatment planning system. The dotted line represents the moving range (4 cm) of the Ir-192. Right panel: absorption dose as a function of distance from the Ir-192 source on the X-axis. Absorption dose was determined using a TLD sheet; irradiation was performed in such a manner as to obtain 6 Gy at the point A, according to the simulation

We used the microSelectron-HDR system (Nucletron, Veenendaal, The Netherlands) equipped with Ir-192 as a radiation source. Twenty-four hours after HeLa-Fucci cells (5 × 10^4^ cells) were seeded on a 35-mm glass-bottom dish (Asahi Glass, Tokyo, Japan), the cells were irradiated as shown in Fig. 1a. A 6 Fr catheter was used as an applicator and placed on the same plane as the dish. The applicator was positioned to cover a span of 40 mm, so that the whole area of the 35-mm dish was included within the effective range of irradiation. The radiation dose was prescribed to be 6 Gy at the hypothetical treatment point A, 20 mm away in a direction perpendicular to the long axis of the applicator (Fig. 1b). Because irradiation was performed in a water bath at 37 °C, the 35-mm dish was coated with a Parafilm sheet (Bemis, Neenah, WI, USA). Dose distribution was simulated by the treatment planning system (Oncentra Brachy) (Elekta, Stockholm, Sweden) and some iso-dose curves were obtained. Independently, we actually measured absorption dose by thermoluminescent dosimetry (TLD) [12]. Briefly, after irradiation on the TLD plate to obtain 6 Gy at the point A, according to the simulation, the plate was heated at 275 °C for 480 s. Luminescence intensity was quantitated, and absorption dose was expressed as a function of distance from the radiation source on the X-axis.

### Irradiation under hypoxic conditions

Using the BIONIX-1 hypoxic culture kit (Sugiyama-Gen, Tokyo, Japan), HeLa-Fucci cells were made hypoxic (pO_2_ < 0.1 %) as described previously [13] and irradiated as described above. Briefly, 24 h after cells (5 × 10^4^ cells) were seeded on a 35-mm glass-bottom dish, the medium was replaced by 300 μl of fresh growth medium to quickly equilibrate the gas and liquid phases. The dish was placed in an AnaeroPouch (Mitsubishi Gas Chemical, Tokyo, Japan) along with an AnaeroPack-Anaero 5 % oxygen absorber (Mitsubishi Gas Chemical) and an OXY-1 oxygen monitor (JIKCO, Tokyo, Japan), and the pouch was sealed. Irradiation was performed 2 h after verifying an oxygen monitor reading of 0 %, which confirms pO_2_ < 0.1 % in the gas phase, to ensure equilibration between the gas and liquid phases. The oxygen enhancement ratio (OER) obtained from dose–cell survival curves (at a surviving fraction of 0.1) was ~2.1. Time-lapse imaging was performed under normoxic conditions.

### Acquisition of low-power images

For acquisition of low-power images, cells grown on dishes were fixed in 4 % paraformaldehyde 12, 24, 48, or 72 h after irradiation under normoxic conditions. A BIOREVO BZ-9000 fluorescence microscope (KEYENCE, Osaka, Japan) was equipped with a low-power objective lens (×10), and fluorescence images were acquired in a field measuring 15 mm × 3 mm in order to include cells located 5 to 20 mm from the radiation source.

### Time-lapse imaging after irradiation

Time-lapse imaging was performed immediately after irradiation. Fluorescence images were acquired on a BIOREVO BZ-9000 fluorescence microscope (KEYENCE) equipped with a high-power objective lens (×40) in an incubation chamber kept at 37 °C in a 5 % CO_2_ humidified atmosphere. Fluorescence images were taken every 2 h until 72 h after irradiation. The field size was 700 μm (length) × 500 μm (width); at least three fields were collected from a total of about 90 cells at each point 5–20 mm from the radiation source. Several parameters were analyzed: the first green phase duration following irradiation (FGPD), reflecting the duration of G2 arrest; the number of entries into M phase; the proportion of cells undergoing mitotic catastrophe; and cell-cycle phase at the time of cell death.

### Dose–cell survival curves

Dose–cell survival curves were obtained from colony forming assays. Exponentially growing HeLa-Fucci cells were irradiated with varying doses under normoxia or hypoxia, generated as described above, using an RX-650 Cabinet X-radiator system (Faxitron, Lincolnshire, IL, USA) at a dose rate of 0.8 Gy/min (130 kVp, 5 mA, 0.5 mm Al filtration). After irradiation, an appropriate number of cells were seeded in 60-mm dishes and incubated for 10 days. Clonogenic survival was determined by counting crystal violet–stained colonies consisting of more than 50 cells.

#### Cell sorting and flow-cytometric analysis

Cell sorting and flow-cytometric analysis was performed as described previously [14]. Briefly, exponentially growing HeLa-Fucci cells were sorted on a MoFlo XDP (Beckman Coulter, Brea, CA, USA) according to their Fucci fluorescence. The red and green fractions were irradiated at a dose of 10 Gy, and 30 min or 3 h after irradiation, the cells were fixed in 4 % paraformaldehyde for 30 min. After staining with an anti-phospho-histone H2AX (Ser139) antibody conjugated with Alexa Fluor 647 (1:50; Cell Signaling, Danvers, MA), each sample was analyzed on a FACSCanto II flow cytometer (Becton Dickinson, Franklin Lakes, NJ, USA) using the FlowJo software (Tree Star, Ashland, OR, USA).

### Statistical analysis

Mann–Whitney *U* test or chi-square test was used for statistical determinations. *P* values < 0.05 were considered statistically significant.

## Results

### Dose distribution under our experimental conditions

The experimental conditions are outlined in Fig. 1a. Simulation of dose distribution by the treatment planning system, which is actually used in the clinical setting in our hospital, is depicted in Fig. 1b by some iso-dose curves (left panel). The dose absorption on the X-axis measured by a TLD plate as a function of distance from the radiation source is shown in Fig. 1b (right panel). The actual absorption dose at 5, 10, 15, and 20 mm was 24, 14, 9, and 6 Gy, respectively.

### Temporo-spatial cell-cycle kinetics in low-power field images

**Fig. 2:**
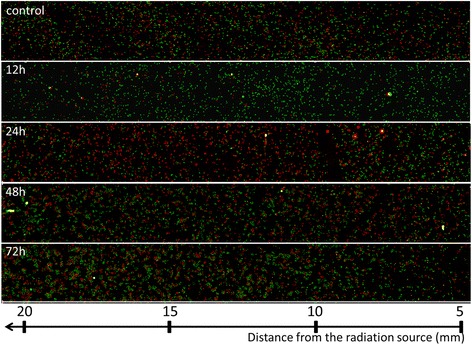
Low-power images of Fucci fluorescence following irradiation. Cells grown on 35-mm dished were irradiated with 6 Gy at point A as shown in Fig. 1, and then fixed at the indicated times after irradiation. Fluorescence images were acquired using a × 10 objective lens at distances ranging from 5 to 20 mm from the Ir-192 source

As a primary goal of this study, we attempted to visualize the cell-cycle kinetics of cells at different distances from the radiation source. For this purpose, we used HeLa cells expressing the Fucci system [9]. In our previous reports using HeLa-Fucci cells, we showed that elongation of the first green phase duration after irradiation (FGPD) and subsequent appearance of red cells perfectly reflects the G2 arrest kinetics following X-irradiation [10, 11]. We reasoned that the fluorescence kinetics could be used to obtain information regarding G2 arrest kinetics as a function of distance from the Ir-192 source. The results are shown in Fig. 2. In general, ~50 % of exponentially growing HeLa-Fucci cells expressed green fluorescence. After irradiation, the proportion of green cells gradually increased, irrespective of the distance from the Ir-195 source up to 20 mm, reaching almost 100 % 12 h after irradiation of cells within the field. However, in cells more distant from the Ir-192 source, red cells (representing cells entering G1 phase after release from G2 arrest) began to appear, and the proportion of green cells gradually decreased. The red fluorescence wave reached 7–8 mm from the radiation source 24 h after irradiation, as shown in the middle panel. This result indicated that G2 arrest occurred similarly in cells up to hypothetical point A, and that release from G2 arrest strongly depended on distance from the Ir-192 source. Forty-eight hours after irradiation, red cells appeared even 5 mm from the source. Cell density was clearly lower closer to the Ir-192 source, and higher at more distant positions, 72 h after irradiation. Thus, we succeeded for the first time in visualizing the cell-cycle kinetics in cells located at different distances from an Ir-192 HDR-RALS.

### Closer analysis using time-lapse imaging of HeLa-Fucci cells 5 or 20 mm from the radiation source

**Fig. 3:**
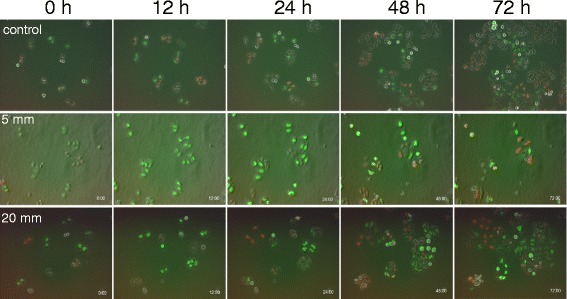
High-power time-lapse images of Fucci fluorescence following irradiation. Cells grown on 35-mm dishes were irradiated with 6 Gy at point A as shown in Fig. 1, and the dishes were transferred to an observation chamber. Fluorescence images were acquired every two hours until 72 h after irradiation at positions 5 and 20 mm from the Ir-192 source. Unirradiated cells were also observed as a control

**Fig. 4:**
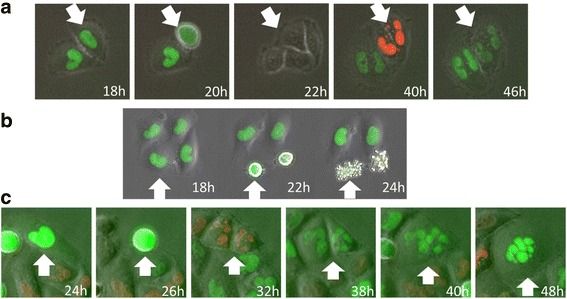
Representative images of mitotic catastrophe. **a** Micronuclei. After mitosis, micronuclei were observed in both daughter cells, and cell fusion occurred. **b** Apoptosis during mitosis. After cells entered mitosis, cells underwent apoptosis during mitosis. **c** Multiple nuclei. After mitosis, multiple nuclei were formed in both daughter cells and cell fusion occurred, resulting in a giant cell

Next, we more closely analyzed the cell-cycle kinetics using time-lapse imaging at higher magnification. The results of time-lapse imaging at distances of 5 and 20 mm from the Ir-192 source are shown in Fig. 3, including images of unirradiated cells used as a control. Qualitatively, we could see that FGPD was elongated in cells at 5 mm, and that some of these cells subsequently became red, but no regrowth was observed. On the other hand, cells at 20 mm also increased the proportion of green cells and began to regrow, as reflected by the appearance of red cells. Various types of mitotic catastrophe, which occurs due to incomplete or abnormal mitosis, were observed, including micronuclei (Fig. 4a), apoptosis during mitosis (Fig. 4b), and multiple nuclei in a single cell (Fig. 4c).

**Fig. 5:**
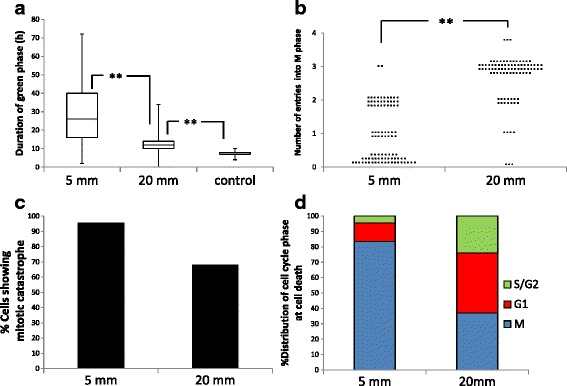
Quantitative analysis of indices related to cell-cycle kinetics following irradiation. **a** FGPD for cells at a distance 5 and 20 mm from the Ir-192 source. Control: without irradiation. Data are represented as box-and-whisker plots showing the full range, 25–75 % interquartile range (*box*), and median (*bar*) of about 90 cells for each group. **, *p* < 0.01. **b** Number of entries into M phase, measured at 5 and 20 mm during the observation period. Data are represented as beeswarm plots from 90 cells for each group. **, *p* < 0.01. **c** Proportion of cells undergoing mitotic catastrophe at the indicated distances from the radiation source, expressed as percentages of all cells, including examples as shown in Fig. 4. **d** Distribution of cell-cycle phases at the time of cell death. Ratios of cell-cycle phases at cell death were determined and expressed as percentages of all dead cells (66 cells at 5 mm; 54 cells at 20 mm)

Next, we performed quantitative analysis of indices related to cell-cycle kinetics (Fig. 5). FGPD was significantly longer in cells at a distance of 5 mm (median: 26 h) than at 20 mm (median: 12 h) from the Ir-192 source (Fig. 5a). The number of entries into M phase up to 72 h after irradiation also differed significantly between cells 5 and 20 mm from the source (Fig. 5b). The percentage of cells undergoing mitotic catastrophe was greater than 65 % at both positions. The distribution of cell-cycle phases at the time of cell death is shown in Fig. 5d. At 5 mm, more than 80 % of cells died during mitosis, whereas at 20 mm, G1 was the most frequent phase (~40 %) in cells that died.

### Differential responses in cells irradiated in green and red phases

**Fig. 6:**
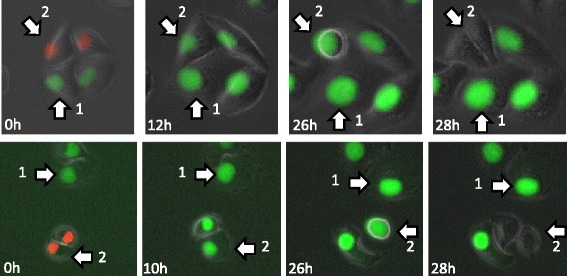
Cells irradiated in red phase enter M phase faster than cells irradiated in green phase. Cell #1, irradiated in green phase; Cell#2, irradiated in red phase. Round cells represent mitosis. Cells were observed at a distance of 5 mm from the Ir-192 source

Previously, in order to analyze the effect of cell-cycle phase at irradiation, it was necessary to isolate cells in specific phases. However, the Fucci system allowed us to obtain this information without using isolated and synchronized populations. Time-lapse imaging revealed that at 5 mm, cells irradiated in red phase entered M phase, whereas those irradiated in green phase stayed green (Fig. 6). This result indicates that the red cells observed at 5 mm 48 h after irradiation (Fig. 2) mostly arose from cells irradiated in red phase. On the other hand, very few examples of this effect were observed at 20 mm (data not shown).

**Fig. 7:**
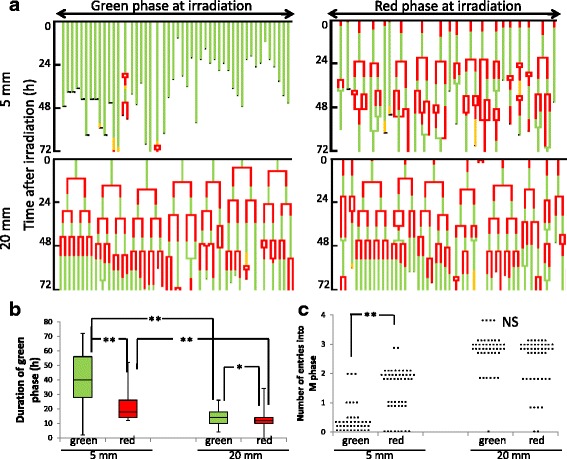
Differential response in cells irradiated in green and red phase. **a** Pedigree analysis for cells irradiated in green and red phase at 5 and 20 mm, analyzed separately. Green bars: green phase in Fucci; red bars: red phase in Fucci; yellow bars: cells abnormally expressing red and green fluorescence at mitosis. **b** FGPD in cells irradiated in green or red phase at 5 or 20 mm. Data are represented as box-and-whisker plots showing the full range, 25–75 % interquartile range (*box*), and median (*bar*) of 45 cells for each group. *, *P* < 0.05; **. *P* < 0.01. **c** Number of entries into M phase in cells irradiated in green or red phase at 5 or 20 mm. Data are represented as beeswarm plots from 90 cells for each group; the 5-mm group consists of 41 green cells and 49 red cells, and the 20-mm group consists of 46 green cells and 44 red cells. **, *P* < 0.01; NS, not significant

This phenomenon suggested that the differences in FGPD depended on whether the cells at 5 mm were irradiated in the green or red phase. Therefore, we classified these cells into two groups according to their fluorescence colors at the time of irradiation, and analyzed them using pedigrees obtained from time-lapse images (Fig. 7a). The median FGPDs for cells irradiated in green and red phase at 5 mm were 40 and 18 h (*p* < 0.01), respectively, whereas those for cells at 20 mm were 14 and 12 h (*p* < 0.05), respectively (Fig. 7b). Because this difference was longer than the normal red phase duration (~8 h) [15] for cells at 5 mm, the phenomenon described above could be due to cells irradiated in red phase entering M phase faster than those irradiated in green phase. On the other hand, such cases were not clearly observed at 20 mm, because the difference was much smaller than ~8 h. We also analyzed the number of entries into M phase as a function of fluorescence color at irradiation. As expected, we observed a marked difference between the phases in cells irradiated at 5 mm (Fig. 7c).

### Responses in cells after irradiation under hypoxic conditions

**Fig. 8:**
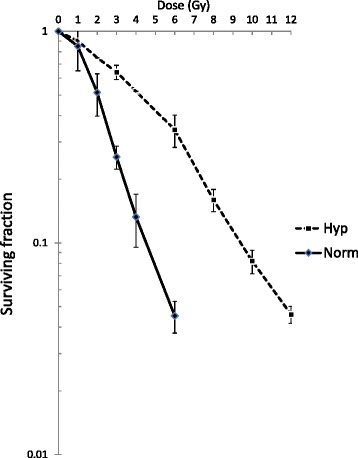
Dose–cell survival curves after X-irradiation under normoxia or hypoxia. Cells were X-irradiated under normoxia or hypoxia and subjected to colony forming assays. Data are represented as means ± S.D. of values obtained from triplicate determinations

**Fig. 9:**
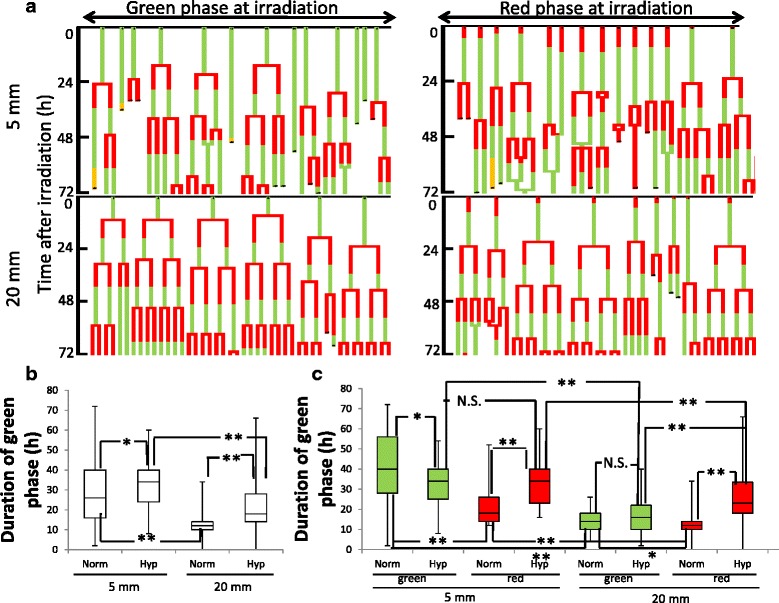
Response to irradiation under hypoxic conditions. **a** Pedigree analysis of cells irradiated in green or red phase at 5 or 20 mm under hypoxia, analyzed separately. Green bars: green phase in Fucci; red bars: red phase in Fucci; yellow bars: cells abnormally expressing red and green fluorescence at mitosis. **b** FGPD of cells irradiated under hypoxia at 5 or 20 mm. Data for cells irradiated under normoxia were derived from Fig. 5a for comparison. Data are represented as box-and-whisker plots, as described in Fig. 7b. Norm, cells irradiated under normoxia; Hyp, cells irradiated under hypoxia. *, *P* < 0.05; **. *P* < 0.01. **c** FGPD of cells irradiated in green or red phase under hypoxia at 5 or 20 mm. Data for cells irradiated under normoxia were derived from Fig. 7b for comparison. Data are represented as box-and-whisker plots, as described in Fig. 7b. Norm, cells irradiated under normoxia; Hyp, cells irradiated under hypoxia. *, *P* < 0.05; **. *P* < 0.01; N.S., not significant

Under in vivo conditions, hypoxic fractions necessarily exist, and such cells are thought to be responsible for tumor recurrence after radiotherapy for cervical cancer [16, 17]. Therefore, we next examined cell-cycle kinetics following irradiation under hypoxic conditions. Under the present hypoxic conditions, the oxygen enhancement ratio (OER) was 2.1 at a surviving fraction of 0.1 obtained from dose-survival curves following varying doses of X-irradiation (Fig. 8). Judging from the surviving fraction (0.4–0.5) at 6 Gy under hypoxia (a dose corresponding to the position at point A), not a few cells could be expected to survive. Pedigree analysis was performed as shown in Fig. 7a except that cells were irradiated under hypoxia (Fig. 9a). Again, FGPD was dependent on distance when cells that were red or green at the time of irradiation were analyzed together; however, FGPD was significantly longer at each distance when cells were irradiated under hypoxia (Fig. 9b).

When hypoxic cells that were red or green at the time of irradiation were analyzed separately, the same dependence on distance was observed again. Unexpectedly, we found that the elongation after irradiation under hypoxia observed in Fig. 9b was mainly due to cells irradiated in red phase (Fig. 9c). Interestingly, after irradiation under hypoxia, the dependence on cell-cycle phase observed under normoxia was lost at 5 mm, whereas the opposite dependence was observed at 20 mm, where cells irradiated in red phase exhibited a longer FGPD than those irradiated in green phase (Fig. 9c). Taken together, these data show that potentially surviving hypoxic cells especially arising from positions around point A exhibited G2 arrest kinetics distinct from those of normoxic cells destined to be eradicated.

## Discussion

Using the Fucci system, we were able for the first time to simultaneously visualize the cell-cycle kinetics in HeLa cells at different distances (5–20 mm) relative to the radiation source following irradiation by Ir-192 HDR-RALS. Our major findings are summarized as follows; 1) cells in this area exhibited similar G2 arrest kinetics up to 12 h after irradiation; 2) more distant cells gradually began to be released from G2 arrest; 3) cells at 5 mm had a significantly longer FGPD than those at 20 mm; 4) under normoxic conditions, cells irradiated in the green phase had a longer FGPD than those irradiated in red phase, and 5) under hypoxic conditions, cells exhibited the opposite dependence on cell-cycle phase at the time of irradiation, especially in cells arising from positions around point A.

The reason that cells within the observed area accumulated in the green phase with similar kinetics up to 12 h is that the time required to reach G2 arrest is not influenced by the degree of DNA damage. In the Fucci system, cells in S and G2 phases exhibit the same green fluorescence; therefore, we can say that red phase duration was not increased even very close to the radiation source. Presumably, at greater distances (>20 mm), the degree of accumulation in green phase gradually decreased. Distance exerted a clear and significant influence on FGPD. This observation indicates that the duration of G2 arrest was strongly dependent on distance from the Ir-192 source, demonstrating that it was primarily influenced by absorption dose. Previous work showed that the duration of G2 arrest is proportional to absorption dose [15, 18]; however, in this study, the use of HeLa-Fucci cells allowed us to clearly temporo-spatially visualize this phenomenon, even in the steep dose gradient of an Ir-192 HDR-RALS.

We can discuss the difference in FGPD from the viewpoint of distance from the radiation source, i.e., the absorption dose. We speculated that this could be attributed to the degree of DNA damage, which is proportional to the absorption dose; however, it should be noted that under normoxia, FGPD at 5 mm was remarkably affected by cell-cycle phase (i.e., green vs red) at the time of irradiation. Indeed, the FGPD was much longer when cells were irradiated in green phase. To explain the phenomenon, we must consider other secondary factors. DNA double-strand breaks (DSBs) are thought to be the most crucial determinants of the DNA damage response, including cell-cycle checkpoints [19–21]. DSBs are repaired through two distinct pathways, non-homologous end joining (NHEJ) and homologous recombination. The former occurs in all cell-cycle phases, whereas the latter is restricted to S and G2 phases [22]. Therefore, in the Fucci system, only NHEJ occurs in red phase, and both pathways are activated in green phase. Karanam et al. reported that when cells are irradiated in G1 phase, DSB repair via the NHEJ pathway is completed within a short period of time, whereas when cells are irradiated in S/G2 phases, it takes longer to perform repair because more time is required to choose one of the two pathways, leading to an elongated G2 arrest [23]. As a matter of fact, our preliminary study showed that quantitative flow-cytometric analysis of γH2AX, a marker of DSBs, after cell sorting revealed a more rapid reduction of the mean fluorescence intensity after irradiation (30 min → 3 h) in cells irradiated in red phase (mean ± SD: 739 ± 26 → 380 ± 14) than in cells irradiated in green phase (1703 ± 95 → 2044 ± 36). Collectively, we can say that the time required for DSB repair is proportional to the number of DSBs induced by irradiation, which in turn corresponds to the absorption dose; this explains our observation that FGPD depended on absorption dose. However, FGPD could be secondarily affected by the DSB repair rate, which is determined by whether cells are irradiated in G1 or S/G2 phase.

Considering that the radiation doses given under normoxic conditions described above ultimately eradicate most HeLa-Fucci cells (Fig. 8), one could argue that our findings are not clinically interesting. Given that hypoxic cells exist within solid tumors, such surviving cells could contribute to recurrence after irradiation. Indeed, many studies have shown that cervical cancers with higher hypoxic fractions are more likely to recur after radiotherapy [16, 17]. Under our experimental conditions, many hypoxic cells around point A, receiving ~6 Gy, were likely to survive (surviving fraction, 0.4–0.5; Fig. 8). Therefore, we next examined cell-cycle kinetics after irradiation under hypoxic conditions. Our results were unexpected because the dependence on cell-cycle phase at the time of irradiation, as reflected in elongation of FGPD, differed between cells irradiated under normoxia and hypoxia, especially around point A. Despite some controversial reports [24], it is generally accepted that under hypoxia, DSB yield after irradiation is lower and its repair is slower [25]. If G2 arrest was determined only by DSB-associated events, then the elongation of G2 arrest could be explained by the fact that DSB repair is slower after irradiation under hypoxia. Furthermore, DSB generation due to reoxygenation after hypoxia might also be involved [14, 26]. However, our observation that G2 arrest is enhanced when cells were irradiated in red phase could not be explained by the notion proposed by Karanam et al. [23], as described above. The yield of DNA-protein cross-linking is enhanced by irradiation under hypoxia, and such cross-links are repaired much more slowly than DSBs [27]. Although the dependence on cell-cycle phase at the time of irradiation remains largely unclear, other types of DNA damage that preferentially occur after irradiation under hypoxia might influence G2 arrest. Taken together, the results of our temporo-spatial study suggest that cells with a high possibility of recurrence, especially those arising from hypoxic tumor cells around point A, exhibit different G2 arrest kinetics than those arising from normoxic cells destined to be eradicated.

There are two types of hypoxia within solid tumors; acute and chronic. The former is the result of the perfusion limit of tumor vessels, caused by their repeated occlusion and re-opening, whereas the latter is the result of the diffusion limit and reoxygenation could occur after irradiation [28]. Our experimental hypoxic conditions may not exactly reflect the quite complicated hypoxia/ reoxygenation kinetics in vivo; however, the present study would shed additional light on the effect of irradiation under hypoxia on the DNA damage response.

## Conclusions

We analyzed cell-cycle kinetics of HeLa-Fucci cells irradiated in monolayer cultures by Ir-192 HDR-RALS. Our findings visualized G2 arrest kinetics which depend on distance from the source and cell-cycle phase at the time of irradiation. The latter dependence differed between irradiation under nomoxia and hypoxia, and the potentially surviving hypoxic cells exhibited unique G2 arrest kinetics. This study may be insufficient to predict actual events in a more complex clinical setting. Further studies using in vivo solid tumors are required to simulate the real radioresponse following irradiation with Ir-192 HDR-RALS.

## Abbreviations

DSB, DNA double strand break; FGPD, first green phase duration; Fucci, fluorescence ubiquitination-based cell cycle indicator; HDR-RALS, high dose-rate remote afterloading system; HPV, human papilloma virus; NHEJ, non-homologous end joining; TLD, thermoluminescent dosimetry

## Additional files

Additional file 1: Table S1. Datasets for Fig. 5. (XLSX 10 kb) Additional file 2: Table S2. Datasets for Fig. 7. (XLSX 10 kb) Additional file 3: Table S3. Datasets for Fig. 8. (XLSX 9 kb) Additional file 4: Table S4. Datasets for Fig. 9. (XLSX 9 kb)
